# Performance dataset on a nearly zero-energy office building in temperate oceanic climate based on field measurements

**DOI:** 10.1016/j.dib.2023.109217

**Published:** 2023-05-09

**Authors:** Deepak Amaripadath, Shady Attia

**Affiliations:** Sustainable Building Design Lab, Department of UEE, Faculty of Applied Sciences, University of Liege, 4000 Liege, Belgium

**Keywords:** Building performance, Thermal discomfort, Energy efficiency, Greenhouse gas emissions, HVAC, Overheating, Overcooling

## Abstract

This dataset was developed between May 2018 and April 2019 to evaluate the building performance of a nearly zero-energy office building in a temperate oceanic climate. This dataset refers to the research paper titled “Performance evaluation of a nearly zero-energy office building in temperate oceanic climate based on field measurements”. The data provides the evaluation of air temperature, energy use, and greenhouse gas emissions from the reference building in Brussels, Belgium. The importance of the dataset lies in its unique data collection approach that provides detailed information on electricity and natural gas use, along with indoor and outdoor ambient temperature values. The methodology involves compiling and refining the data from the energy management system installed in Clinic Saint-Pierre, Brussels, Belgium. Hence, the data is unique and not available on any other public platforms. The methodology followed in this paper to produce the data was an observational approach focused on field measurements of air temperature and energy performance. This data paper will be beneficial for scientists working on the implementation of thermal comfort strategies and energy efficiency measures toward energy-neutral buildings while accounting for the performance gaps.

**Specifications Table**

**Table:** 

Subject:	Engineering
Specific subject area:	Building energy performance
Type of data:	Tables – Excel files (.xlsx)
How the data were acquired:	Case-study building: Clinic Saint-Pierre, Brussels, BelgiumMeasurement devices: Air temperature [°C] – Temperature loggersEnergy use [kWh] – Energy metersMeasurement period: May 2018 to April 2019Measurement frequency: Air temperature [°C] – hourly valuesEnergy use [kWh] – monthly values Monitoring process: Continuous Available data: 1. Hourly indoor and outdoor temperature (°C) 2. Monthly cooling and heating energy use (kWh/m^2^) 3. Monthly cooling and heating GHG emissions (kg.CO_2_e/m^2^)
Data format:	Raw – Hourly indoor and outdoor ambient temperature [°C] Monthly cooling and heating system energy use (kWh/m^2^) Analyzed – Monthly cooling and heating GHG emissions (kg.CO_2_e/m^2^).
Description of data collection:	Data on the building performance in terms of ambient temperature, and site energy use in the reference nearly zero-energy office building, Clinic Saint-Pierre were collected. Hourly indoor ambient temperature values were measured using temperature loggers in five different measurement zones, which were selected considering the façade orientation and purpose of the building space. Monthly energy use data were measured using energy meters in the building. The data were then extracted from the building energy management system for the time frame from May 2018 to April 2019. The monthly cooling and heating GHG emissions were calculated using the monthly energy used data.
Data source location:	Case-study building: Clinic Saint-Pierre City: Brussels Country: Belgium GPS coordinates: 50° 40′ 04.67" N and 04° 33" 39.68" E Elevation: 112 m Climate zone: Temperate oceanic climate (Cfb) Building type: Commercial building Building certification: Passive House certification via Passive House Planning Package (PHPP) & dynamic simulations
Data accessibility:	Repository name: Harvard Dataverse Data identification number: 10.7910/DVN/NLEAKA Direct URL to data: https://dataverse.harvard.edu/dataset.xhtml?persistentId=doi%3A10.7910%2FDVN%2FNLEAKA Reference: D. Amaripadath and S. Attia, “Field measurement dataset of a nearly zero-energy office building in temperate oceanic climate,” Harvard Dataverse, Cambridge, USA, 2022. doi: 10.7910/DVN/NLEAKA[2]. License: CC0 1.0 Universal Public Domain Dedication
Related research article:	D. Amaripadath, M. Velickovic, and S. Attia, “Performance evaluation of a nearly zero-energy office building in temperate oceanic climate based on field measurement,” *Energies*, vol. 15, p. 6755, Sep. 2022. doi: 10.3390/en15186755[1].

## Value of the Data

• Field measurement data is significant to calibrate buildings in terms of essential parameters, such as air temperature, energy use, and GHG emissions, among others. In addition, this dataset can be used to identify problems in building operations and to develop alternative means for improving building efficiency. Field measurement data is usually the final verification indicator for complex design and construction process involved in the building sector.
• This data accurately and precisely represents the operational characteristics of a nearly zero-energy office building in temperate oceanic climate (Cfb) according to the Köppen-Geiger climate classification [3], [4], [5]. Comparability of the data, in terms of that it can contribute towards common analysis and comparison with other buildings in similar climates adds to the value of this dataset [6]. Sample collection on multiple zones enables a multizonal and time-integrated thermal discomfort analysis.
• The data sheds light on the implications of nearly zero-energy office building implementations in Brussels, Belgium. This data makes an important contribution to the new body of knowledge database by providing a clear picture of building performance evaluation in temperate oceanic climates (Cfb) according to the Köppen-Geiger climate classification [3], [4], [5]. This climate is especially prevalent in Western European cities, such as Amsterdam, London, and Paris [7].
• The data is useful for the building community, which includes facility managers, building owners, energy engineers, building engineers, energy modelers, HVAC engineers and designers, who want to design, build, and operate future energy-efficient and carbon-neutral buildings. More importantly, the data enables scientists to study the energy performance gaps and to create energy policies that translate design data and code requirements that are in line with EU regulations and roadmaps that aim to reduce the emissions by 80% by the 2050s [8].

## 1 Objective

The main objective of this dataset was to add to the existing knowledge base of real building performance data. Anything that can be measured, can be managed and improved. Hence, this dataset can contribute to better building operation, planning, and performance. Field measurement data is indispensable to provide a clear and compelling case for the effective use of energy resources. The dataset is related to the research article titled “Performance evaluation of a nearly zero-energy office building in temperate oceanic climate based on field measurement,” that evaluates thermal comfort with respect to EN 16798-1 [9]. Publishing the data article and the dataset will help building scientists and engineers to evaluate thermal comfort with respect to standards like ISO 17772 [10] and ASHRAE 55 [11]. In addition, outdoor air temperature data can be used to assess heatwave events and building performance during these extreme events. The energy use and GHG emission datasets from the building were created to evaluate how the existing building performs and effects the environment. This data is important in terms of climate change mitigation.

## 2 Data Description

Data presented in this article include raw and analyzed data collected from a nearly zero-energy office building in Brussels, Belgium, which is located in the temperate oceanic climate zone (Cfb) according to the Köppen-Geiger climate classification [4,5]. The dataset associated with this article contains three excel files:

1. Air Temperature Data.xlsx
  a. Column 1 describes the date of measurement;
  b. Column 2 describes the time of measurement;
  c. Column 3 describes the hourly outdoor ambient temperature in °C;
  d. Column 4 describes the hourly indoor ambient temperature in °C and is subdivided into five measurement zones.
2. Monthly Energy Use.xlsx
  a. Column 1 describes the month and year of measurement;
  b. Column 2 describes the monthly cooling energy use per square meter in kWh/m^2^;
  c. Column 3 describes the monthly heating energy use per square meter in kWh/m^2^.
3. Monthly cooling and heating GHG emissions (kg.CO_2_e/m^2^)
  a. Column 1 describes the month and year of measurement;
  b. Column 2 describes the monthly cooling GHG emissions per square meter in Kg.CO_2_e/m^2^.a;
  c. Column 3 describes the monthly heating GHG emissions per square meter in Kg.CO_2_e/m^2^.a.

**Fig. 1:**
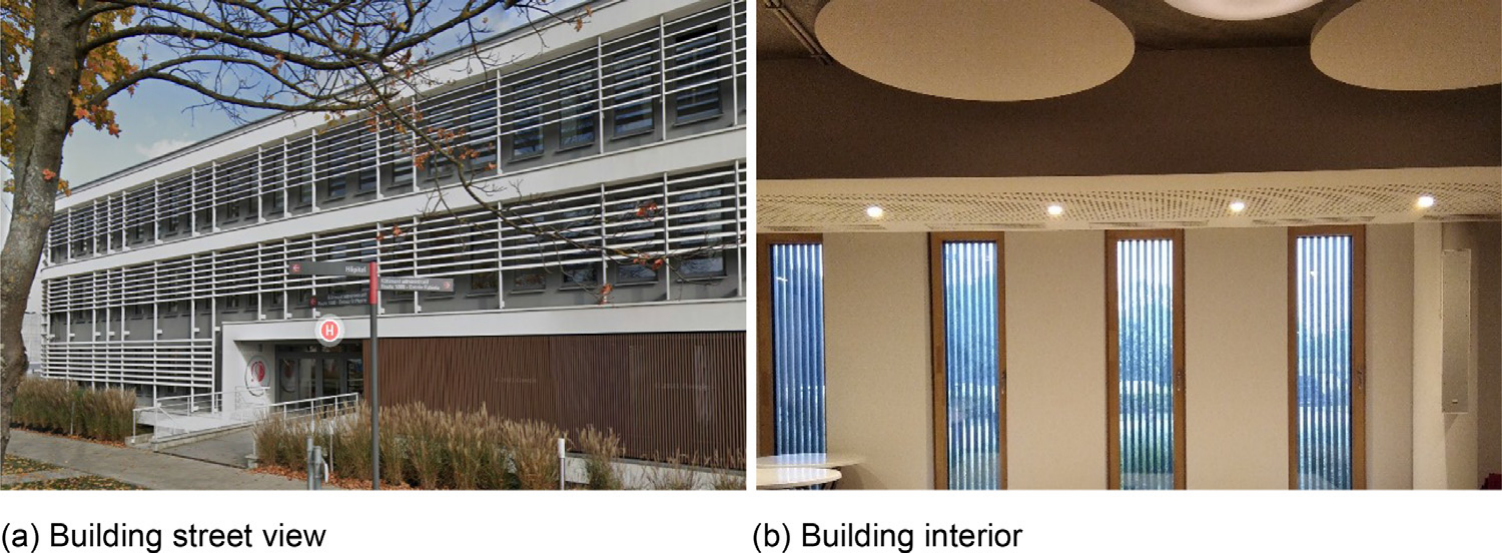
Clinic Saint-Pierre, Brussels, Belgium - a nearly zero-energy office building located in temperate oceanic climate (Cfb).

The street view and interior of the reference building are shown in Fig. 1(a) and Fig. 1(b), respectively. The horizontal slats used for solar shading in the building are visible in Fig. 1(a). The dataset from the monitoring campaign is deposited in an open access repository [2]. This dataset includes three different Excel files in .xlsx format that represents multizonal hourly indoor and outdoor ambient temperature values (°C), monthly cooling and heating energy use (kWh/m^2^), and monthly cooling and heating energy emissions (kg.CO_2_e/m^2^).

The collected dataset from the reference building can be used to evaluate the following parameters:

1. Air Temperature Data.xlsx can be used for Indoor thermal comfort evaluation as per EN 16798-1 [9], ISO 17772 [10], ASHRAE 55 [11], etc., and time-integrated overheating [12] and overcooling [13] evaluation.
2. Monthly Energy Use.xlsx can be used for energy performance gap analysis and cooling and heating system efficiency studies.
3. Monthly GHG Emissions.xlsx can be used to evaluate the building GHG emissions from cooling and heating systems.

## 3 Experimental Design, Materials and Methods

The European Union (EU) aims to reach carbon neutrality in the building sector by 2050. The Fit to 55 legislative package aims to achieve carbon-neutral buildings across Europe by 2030 [14]. The EU uses the Energy Performance of Building Directive (EPBD) as a tool to convert carbon neutrality targets into technical plans and requirements for newly built and existing buildings [15]. The first step toward Net Zero Energy Buildings (NZEBs) and nearly Zero Carbon Buildings (nZCBs) is the development of nZEBs [16]. Since 2018, Western Europe [17] and Southern Europe [18] have reported numerous advancements in nZEBs. However, regarding actual building operation and performance, much information is not available.

Quantitative research is primarily an observational methodology as it seeks to understand building characteristics through field measurements. Therefore, it continues to be the most effective exploratory scientific methodology that offers insightful analyses and interpretations of building performance. The prolonged yearlong engagement from May 2018 to April 2019 allowed for a deeper understanding of the performance characteristics of the reference building in Brussels, Belgium. Understanding the underlying causes of thermal comfort and energy use in buildings will help to validate the performance.

The different measurement zones used for indoor ambient temperature (°C) data and energy use [kWh] is available in [1]. These measurement zones were selected considering the façade orientation and purpose of the building space. The measurement locations are as follows: (i) R77 is an office room with a northeast façade, (ii) R81 is a meeting room without an exposed façade, (iii) R92 is an office room with a southwest façade, (iv) R101 is an office room with a southeast façade, and (iv) R102 is an office room with a southeast façade.

This dataset was extracted from the energy management system maintained by Equans in the reference net zero-energy building, Clinic Saint-Pierre in Brussels, Belgium. The shared field measurement data include refined and processed data in excel files (.xlsx format). The preparation and compilation of the dataset were performed on a cutting-edge workstation at the Sustainable Building Design Lab, Super COmputeR ProcessIng wOrkstatioN (SCORPION), which has a processor with 6 cores, 128 threads, and a 256 MB cache for computing power and performance. This is in addition to a 128 GB of Random Access Memory (RAM) and a 24 GB graphics card capable of handling most scientific applications. The dataset described in this paper was acquired through a yearlong field measurements from the reference building, and the dataset satisfies following criteria [19]:

1. The data format is available in excel format (.xlsx), which is very widely used for data analysis.
2. The data is generated through field measurements, which increases its applicability for real case scenarios in building performance. The dataset is well described including the measurement locations in Section 2, well documented, and available through an open access platform, Harvard Dataverse [2].
3. The utility of data for future studies are also explained in this paper, including time-integrated thermal discomfort evaluation, energy efficiency evaluation, and performance gap analysis, among others.
4. The potential reusability of the data is evident from the fact that many Western European cities like Amsterdam, Paris, etc., share a similar climate as Brussels, where the reference building is located.
5. In addition, the dataset is valuable due to growing interest in performance gap research [20] and Europe's journey towards carbon neutrality [8].
6. This dataset article was prepared according to the Data in Brief journal criteria and template, describing the data format, data acquisition, data analysis, data accessibility, data utility, and data reusability. The associated research article [1] is also mentioned in this paper.

## Ethics Statements

The above work does not contain information from human subjects, animal experiments, or data collected from social media platforms.

## CRediT authorship contribution statement

**Deepak Amaripadath:** Conceptualization, Methodology, Software, Formal analysis, Investigation, Resources, Data curation, Writing – original draft, Visualization, Project administration, Funding acquisition. **Shady Attia:** Conceptualization, Validation, Writing – review & editing, Supervision, Project administration, Funding acquisition.

## Declaration of Competing Interest

The authors declare that financial support was provided by the Walloon Public Service and MK Engineering, Belgium. The funders had no role in the study design, in the collection, analyses, or interpretation of data, in the writing of the manuscript, or in the decision to publish the results.

## Data Availability

Field measurement dataset of a nearly zero-energy office building in temperate oceanic climate (Original data) (Harvard Dataverse).
